# There is no ‘Conundrum’ of InsP_6_

**DOI:** 10.1098/rsob.150181

**Published:** 2015-11-18

**Authors:** Robin F. Irvine, Simon J. Bulley, Miranda S. Wilson, Adolfo Saiardi

**Affiliations:** 1 Department of Pharmacology, University of Cambridge, Tennis Court Road, Cambridge CB2 1PD, UK; 2 Department of Haematology, Cambridge University Hospitals NHS Trust, Hills Road, Cambridge CB2 0QQ, UK; 3 Medical Research Council Laboratory for Molecular Cell Biology, University College London, London, UK

## Abstract

Indirect assays have claimed to quantify phytate (InsP_6_) levels in human biofluids, but these have been based on the initial assumption that InsP_6_ is there, an assumption that our more direct assays disprove. We have shown that InsP_6_ does not and cannot (because of the presence of an active InsP_6_ phosphatase in serum) exist in mammalian serum or urine. Therefore, any physiological effects of dietary InsP_6_ can only be due either to its actions in the gut as a polyvalent cation chelator, or to inositol generated by its dephosphorylation by gut microflora.

We are grateful to Dr Vucenik for bringing up a number of interesting points.

It is true that we have not quantified the dietary intakes of our human donors any more (but also hardly any less) than has been done by those groups claiming that InsP_6_ is present in bodily fluids. As a qualitative observation we should point out that in fact all our donors for ref. [1] do have a regular intake of dietary cereals and indeed, one is a strict vegetarian on a high cereal diet. But it is quantification that reveals this to be a specious issue. The limits of detection in our two relevant publications [1,2] for InsP_6_ in plasma and urine were, respectively, around two and three orders of magnitude lower than the levels claimed to be present by Grases *et al.* [3] in the fluids of experimentally phytate-deprived human subjects. These numbers make the argument that we could not detect any InsP_6_ simply because we chose donors on the ‘wrong’ diet untenable.

So how have those many claims that InsP_6_ is present in body fluids come about? For most of them, the simple answer appears to be that the assays used are indirect and are based entirely on the assumption that InsP_6_ is present in the first place. Thus, for example, Valiente and co-workers [4,5] and Chen and co-workers [6,7] measured organic phosphate remaining after a series of fractionations of urine samples and simply assumed it was due to InsP_6_, as did March *et al*. measuring inorganic phosphate after a similar protocol [8]. Grases co-workers [9] have used extensively a less indirect assay, which, after initial ion chromatography and dephosphorylation by a phytase, measures myo-inositol by mass spectrometry, but nevertheless the assay starts with the assumption that InsP_6_ is there and that this is what they are quantifying. More recently, direct quantification of InsP_6_ in plasma by mass spectrometry has been claimed [10] on the basis that there are peaks in plasma at *m/z* 624 running near where InsP_6_ standards elute in two different HPLC separations [10,11]. But no evidence is presented to show even that these peaks are the same compound, let alone any data to establish firmly that InsP_6_ is present, e.g. a minimal requirement of *m/z* quantified to two decimal places with allowance for C_13_ content or a full disintegration fingerprint (see also [12]). Any quantified misidentification is likely to have a stochastic element to it, and it is noteworthy that Perelló & Grases have stated [11, p. 255]: ‘…we have found some humans and rats having undetectable [InsP_6_], probably depending on their diet or other unknown factors’. In the light of the preceding discussion, we can offer a simpler explanation: the InsP_6_ was never there in the first place.

In contrast to these claims we have, using two entirely independent specific and sensitive assays with quantified spiking recovery, unambiguously shown that InsP_6_ is not present in plasma or urine. This is crucial and central to the whole debate about the actions of dietary InsP_6_, because it means that InsP_6_ never enters the blood. It is only absorbed after being dephosphorylated, principally to inositol (see [1,2] for further discussion). Ironically, the most direct evidence for this lies in Dr Vucenik's own data in experiments examining the fate of radioactive InsP_6_ fed to animals, in which only inositol was detected in the blood [13]. This particular study was, as Dr Vucenik points out in her letter, conducted on mice. However, exactly the same conclusion (i.e. InsP_6_ does not enter the circulation from the gut) is equally clear in her earlier study [14], which she did not cite and which was indeed on rats; does this omission ‘reflect poorly’ on Dr Vucenik's own ‘report and the author's credibility in culling scientific data’?

In short, dietary InsP_6_ can have only two fates: it can stay in the gut, ultimately to be defecated [15], and while it is there it can chelate metal ions to alter their uptake from the gut into the body. This is no ‘straw-man’ and is certainly the most likely explanation for all of the effects of InsP_6_ on cultured cells, which comprise the majority of the reports cited by Dr Vucenik. Alternatively, InsP_6_ can be converted to inositol (principally by the gut microflora [15]) and be taken up as such into the circulation; were any InsP_6_ to get into the blood it would in any case be rapidly dephosphorylated by the phosphatase activity we have shown to be present in human plasma [1].

For animal studies, we have raised the possibility [1,2] that it is the inositol so generated (Vitamin Bh, harmless as far as we know) that is the active mediator of any reported beneficial effects of dietary InsP_6_. We note that most of the websites touting InsP_6_ as a dietary supplement advocate inositol as an important (essential?) co-supplement; that the only human cancer study highlighted as important by Dr Vucenik that we could examine [16] did not administer InsP_6_ alone, but only in conjunction with inositol; and that in the few studies where the separate contributions of inositol and InsP_6_ have been considered, there are data suggesting that it may be the inositol that matters (e.g. fig. 1 of [17]). Moreover, we are not the only ones to suggest this idea. In the Discussion of their paper (on mice) in which InsP_6_ was shown not to enter the blood from the gut [13], Dr Vucenik and her colleagues state: ‘Inositol may be responsible for the antitumor actions observed in both chemopreventitive and efficacy studies of IP_6_ … A question remains as to whether the activity of IP_6_ in animal models can be replicated by administration of inositol alone because only inositol was detected in plasma and tumor after oral gavage’. Precisely.

Finally, returning to InsP_6_ itself, which, incidentally, is officially classified by the FDA as a ‘fake’ cancer cure (http://www.fda.gov/drugs/guidancecomplianceregulatoryinformation/enforcementactivitiesbyfda/ucm171057.htm), our data lead inevitably to the conclusion that while InsP_6_ might impact on the gut environment and thus indirectly on its microflora [2,12], its only plausible direct action on the body will be to inhibit cation uptake from the diet. Although InsP_6_ binds trivalent cations with a higher affinity than divalents [18], it is nevertheless comparatively non-specific in this action. Administering chemicals to the diet to manipulate ion uptake is not unknown in modern medicine; for treatment of iron disorders such as haemochromatosis, as an alternative to injection of Desferral, oral administration of the closely related chelator Deferasirox is now sometimes recommended [19]. But Deferasirox is a highly iron-specific chelator, administered under close medical supervision for a directly iron-related pathology. Recommending unmonitored, widespread administration of InsP_6_ to address a veritable multitude of different pathologies [20] seems to us to be an entirely different matter.

In a well-fed human, where the cation to InsP_6_ ratio in the diet is high, InsP_6_ may very well do no harm (it is, after all, a natural component of our diet) and there is much evidence to support this idea, as argued by Dr Vucenik. But if InsP_6_ is not impacting on cation uptake from the diet to do any harm it is difficult to understand how at exactly the same time it can impact on the same uptake to do good. (See reference [21] for the studies Dr Vucenik requested ‘unequivocally demonstrating the toxicity of pure Ca-Mg-InsP_6_ as it occurs naturally’ in humans with low dietary cation uptake.) In the light of the above discussion and our rigorous data, we stand unreservedly by our original closing statement [1]: ‘…that chronically altering cation absorption from the gut by artificially loading the diet with a non-specific chelator … in the hope that it might impact indirectly on cancer or other pathologies seems highly inadvisable’.
